# Effects of *Bacillus amyloliquefaciens* on Growth Performance, Immune Performance, Antioxidant Capacity, Jejunal Microbiota and Transcriptome of Zi Geese

**DOI:** 10.3390/ani16142243

**Published:** 2026-07-20

**Authors:** Qi Zhang, Yueyan Zhou, Jinqiu Xing, Ying Wang, Yuan Li, Lei Liu, Qiuju Wang

**Affiliations:** Heilongjiang Provincial Key Laboratory of Exploration and Innovative Utilization of White Goose Germplasm Resources in Cold Region, College of Animal Science and Veterinary Medicine, Heilongjiang Bayi Agricultural University, Daqing 163319, China; zhangxiaoqi0405@163.com (Q.Z.); zhouyy0724@163.com (Y.Z.); xjq1261581811@163.com (J.X.); wying18403878608@163.com (Y.W.); 19845976068@163.com (Y.L.); l13225841092@163.com (L.L.)

**Keywords:** *Bacillus amyloliquefaciens*, Zi geese, growth performance, intestinal health, probiotic

## Abstract

With the global ban on antibiotic growth promoters, identifying safe, effective alternatives for poultry production is urgent. Probiotics, particularly *Bacillus amyloliquefaciens* (BA), have shown promise in improving animal health and performance, but research in geese remains limited. This study aimed to evaluate the effects of dietary BA supplementation on growth performance, immune function, antioxidant capacity, intestinal morphology, microbiota, and transcriptome in growing Zi geese. A total of 180 50-day-old male Zi geese were randomly assigned to a control group (basal diet) or an experimental group (basal diet supplemented with 1 × 10^8^ CFU/g BA) for 40 days. Growth performance, serum immune indices, intestinal antioxidant parameters, morphology, jejunal microbiota, and transcriptomic profiles were assessed. BA supplementation significantly increased average daily gain and decreased feed-to-gain ratio (*p* < 0.01). Serum IgA, IgG, IgM, and IL-4 increased, while IL-2, IL-1β, and TNF-α decreased (*p* < 0.01). Intestinal CAT, GSH-Px, and T-AOC increased (CAT in duodenum and jejunum, *p* < 0.01; CAT in ileum, *p* < 0.05; GSH-Px in duodenum and ileum, *p* < 0.01; GSH-Px in jejunum, *p* < 0.05; T-AOC in all segments, *p* < 0.01), and jejunal MDA decreased (*p* < 0.01). Villus height and V/C ratio improved. Jejunal microbial diversity increased, with higher Proteobacteria and lower Pseudomonas and Escherichia–Shigella. Transcriptomic analysis revealed DEGs enriched in calcium signaling, cell junction, and intestinal barrier pathways. Dietary BA at 1 × 10^8^ CFU/g enhances growth, immunity, and intestinal health in Zi geese by improving gut function, remodeling microbiota, and modulating gene expression, supporting its use as a green feed additive.

## Simple Summary

Raising geese without antibiotics is challenging due to the prevalence of stress and intestinal disorders. This study tested whether supplementing feed with a beneficial bacterium, *Bacillus amyloliquefaciens* (BA), could alleviate these issues. We divided 180 young male geese into two groups: a control group receiving a basal diet and an experimental group receiving the same diet supplemented with BA. After 40 days, the BA-supplemented geese grew faster and exhibited improved feed efficiency. They also demonstrated stronger immune responses, enhanced intestinal antioxidant defenses, healthier gut tissue morphology, and a more balanced microbial community with fewer harmful species. Transcriptomic analysis confirmed the upregulation of genes related to gut barrier function. These results suggest that BA serves as a practical, antibiotic-free strategy to improve goose health and productivity, supporting sustainable farming practices.

## 1. Introduction

The global livestock industry is transitioning toward more efficient, sustainable, and environmentally friendly practices. Poultry production plays a critical role in ensuring a stable supply of animal protein and contributing to rural economic development. The Zi goose, a premier indigenous breed in northern China, possesses valuable genetic traits, including adaptability to cold climates and high reproductive performance, making it a significant genetic resource for improving breeding efficiency. However, the expansion of breeding scale and increased stocking densities easily induce stress and intestinal health problems. Following the prohibition of antibiotics, there is a lack of effective replacements. Therefore, probiotics—which are non-toxic, residue-free natural feed additives that improve intestinal microecology—have become ideal substitutes with broad application prospects [1].

The intestinal tract of poultry serves as the primary interface between the host and the external environment, harboring a complex microbial ecosystem that plays pivotal roles in nutrient digestion, immune modulation, and pathogen resistance. In intensive production systems, high stocking densities, environmental stressors, and dietary transitions frequently disrupt intestinal homeostasis, leading to increased susceptibility to enteric diseases and reduced growth efficiency. The prohibition of antibiotic growth promoters has exacerbated these challenges, as producers now lack effective tools to maintain intestinal health and productivity. Probiotics, particularly spore-forming Bacillus species, have emerged as promising alternatives due to their resilience in feed processing, ability to colonize the intestinal tract, and capacity to produce antimicrobial metabolites and digestive enzymes. However, the efficacy of probiotics is highly strain-specific and dose-dependent, and their mechanisms of action in different poultry species remain incompletely understood. Geese, as important waterfowl species with distinct digestive physiology compared with chickens and ducks, have received limited attention in probiotic research, creating a critical knowledge gap in optimizing their intestinal health management.

As an important member of the genus Bacillus, *Bacillus amyloliquefaciens* (BA) is a Gram-positive probiotic that is widely found in soil, plant surfaces, and animal intestines [2]. Its unique biological characteristics make it have multiple application values in livestock and poultry breeding [3,4,5]. Studies have shown that BA can produce various active substances such as bacteriocins and antimicrobial peptides through its own metabolism, effectively inhibit the growth and reproduction of pathogenic bacteria such as Salmonella and Escherichia coli, and reduce the risk of intestinal infection [6]. At the same time, the bacteria can secrete amylase, protease, cellulase, and other digestive enzymes, promote the decomposition and absorption of nutrients such as starch, protein, and dietary fiber in feed, and improve feed utilization [7,8]. Although the beneficial roles of BA in livestock and poultry have been partially confirmed, its application in goose production is relatively limited. In particular, systematic research on growing geese remains scarce. To determine the optimal dietary concentration of BA for growing geese, we conducted a preliminary experiment. Sixty 50-day-old male geese with similar body weight were randomly divided into four groups with three replicates in each group and five geese in each replicate. Geese in the control group were fed a basal diet, and those in the gradient groups were fed the basal diet supplemented with 1 × 10^7^, 1 × 10^8^, and 1 × 10^9^ CFU/g *Bacillus amyloliquefaciens*, respectively. After a 14-day period, geese in the 1 × 10^8^ CFU/g group exhibited the highest average daily gain and the lowest feed conversion ratio (*p* < 0.05), with no adverse reactions observed. Therefore, 1 × 10^8^ CFU/g was selected as the optimal concentration for the formal experiment. Based on this optimal dosage, the present study systematically evaluated the effects of dietary *Bacillus amyloliquefaciens* supplementation on growth performance, serum immunity, intestinal antioxidant capacity, and jejunal microbial community structure in Zi geese. Concurrently, jejunal transcriptome sequencing was performed to identify differentially expressed genes and elucidate the molecular mechanisms underlying intestinal health and antioxidant regulation. This study aimed to verify three key scientific questions: (1) The regulatory effect of 1 × 10^8^ CFU/g BA on growth and intestinal health of growing Zi geese; (2) The microbial and transcriptomic mechanisms of BA regulating intestinal function; (3) The correlation between intestinal microbiota and host immune/antioxidant indices. Finally, it provides a scientific theoretical basis and practical guidance for the rational application of *Bacillus amyloliquefaciens* in the goose breeding industry, thereby providing a scientific basis for the application of BA as a green feed additive, optimizing management practices, and promoting the sustainable development of the goose industry.

## 2. Materials and Methods

### 2.1. Ethics Approval

This experiment was approved by the Science and Technology Ethics Committee of Heilongjiang Bayi Agricultural University, and the approval number was DWKJXY2024019.

### 2.2. Experimental Materials

*Bacillus amyloliquefaciens* used in the experiment was isolated from the intestine of healthy Zi geese by the Key Laboratory of Germplasm Resources Exploration and Innovative Utilization of White Geese in Cold Regions of Heilongjiang Province in the early stage and was preserved in China Microbiological Preservation Center (CGMCC No. 24883). The effective viable count of *Bacillus amyloliquefaciens* preparation was ≥ 1.0 × 10^11^ CFU/g.

### 2.3. Experimental Design and Feeding Management

In the preliminary experiment, sixty 50-day-old male Zi geese with similar body weight were randomly allocated to four groups (3 replicates per group, 5 geese per replicate). The control group received the basal diet, while the treatment groups were supplemented with 1 × 10^7^, 1 × 10^8^, and 1 × 10^9^ CFU/g *B. amyloliquefaciens*, respectively. The pre-experiment lasted for 14 days. Results show that the 1 × 10^8^ CFU/g group exhibited the highest average daily gain (*p* < 0.05) and the lowest feed conversion ratio (*p* < 0.05) among all groups, with no adverse effects such as feed refusal or intestinal abnormalities observed. Therefore, 1 × 10^8^ CFU/g was selected as the optimal concentration for the formal experiment.

A total of 180 50-day-old male geese with the same genetic background and similar body weight were randomly divided into two groups with 6 replicates in each group and 15 geese in each replicate. The control group (CON) was fed a basal diet (Table 1), while the experimental group (BA) was fed the basal diet supplemented with *Bacillus amyloliquefaciens* preparation (viable count ≥ 1.0 × 10^11^ CFU/g). The addition amount was 1 g/kg diet, so that the final content of the bacteria in the diet reached 1.0 × 10^8^ CFU/g. The experiment lasted for 40 days, and the goose house was fully cleaned and disinfected before the start of the experiment. During the experiment, the geese were fed twice daily (7:00 and 16:00). During the whole period, free feeding and drinking water were provided to ensure natural light, and the immunization was carried out according to the normal immunization procedure. The goose house was cleaned regularly to maintain hygiene, and the health status of the geese was observed daily. During the 40-day experimental period, geese were housed in a naturally ventilated shed with a stocking density of 3 birds/m^2^. The environmental temperature ranged from 18 °C to 28 °C, with an average of 23 ± 3 °C. Relative humidity was maintained between 55% and 75% (average: 65 ± 8%). Natural lighting was provided with a photoperiod of approximately 14 L:10 D during the experimental period. Fresh water was available ad libitum via nipple drinkers, and feed was provided in tube feeders. The shed was cleaned daily, and disinfectant footbaths were placed at the entrance.

### 2.4. Sample Collection and Growth Performance Measurement

At 50, 70, and 90 days of age, the geese were fasted for 12 h without water and weighed in replicates. The average daily feed intake (ADFI), average daily gain (ADG), and feed conversion rate (FCR) were calculated. At 90 days of age, six geese per group were randomly selected from different pens (one goose per pen, avoiding siblings) for blood collection, intestinal tissue sampling, and jejunal content collection. The selection criteria included: (1) body weight within ±5% of the group mean to avoid extreme values; (2) absence of clinical signs of disease; and (3) representation of different pens to account for potential pen effects. Samples from individual birds were processed separately and were not pooled. For microbiota analysis, jejunal chyme was collected aseptically from the middle segment of the jejunum and immediately snap-frozen in liquid nitrogen. For transcriptome analysis, approximately 2 cm of jejunal mucosa was scraped using a sterile glass slide, rinsed with PBS, and stored in RNA later at −80 °C. Each sample was processed and analyzed individually, with the individual bird serving as the experimental unit (n = 6 per group). For growth performance measurements, feed intake was recorded daily at the pen level (15 geese per pen), and body weight was measured individually but analyzed as pen averages. The pen was considered the experimental unit for growth performance analysis (n = 6 pens per group).

### 2.5. Determination of Serum Immune Indexes

The ELISA kit provided by Shanghai Yuanju Biotechnology Center (Shanghai, China) was used to determine the levels of immunoglobulin IgA (immunoglobulin A), IgG (immunoglobulin G), IgM (immunoglobulin M) and cytokines IL-1β (interleukin-1β), IL-2 (interleukin IL-2), IL-4 (interleukin IL-4) and TNF-α (tumor necrosis factorα) in the serum of Zi geese in strict accordance with the manufacturer’s instructions (Catalog numbers:YJ920506 for IgA, YJ920507 for IgG, YJ920508 for IgM, YJ966351 for IL-1β, YJ500452 for IL-2, YJ144710 for IL-4, and YJ155021 for TNF-α).

### 2.6. Determination of Intestinal Antioxidant Enzyme-Related Indicators

For the determination of these indices, approximately 0.1 g of intestinal tissue (duodenum, jejunum, and ileum, respectively) was sampled and homogenized with 1 mL of extraction buffer. Tissue samples were collected from the middle segment of each intestinal section, rinsed with ice-cold PBS to remove luminal contents, snap-frozen in liquid nitrogen, and stored at −80 °C until analysis. The homogenate was centrifuged at 12,000 rpm for 10 min at 4 °C, and the supernatant was collected for subsequent assays. All detections were performed using commercial assay kits supplied by Suzhou Grace Biotechnology Co., Ltd. (Suzhou, China).

The activity of SOD (superoxide dismutase) was determined by the WST-8 method based on the scavenging rate of superoxide anions; CAT (catalase) activity was calculated according to the reduction amount of hydrogen peroxide; GSH-Px (glutathione peroxidase) activity was measured by the DTNB method through the oxidation rate of glutathione; T-AOC (total antioxidant capacity) was evaluated via the FRAP method; the content of MDA (malondialdehyde) was determined by the TBA method (Catalog numbers: G0101W for SOD, G0105W for CAT, G0205W for GSH-Px, G0115W for T-AOC, and G0109W for MDA).

### 2.7. Morphological Analysis of Intestinal Tissue

Duodenal, jejunal, and ileal tissues of geese were collected at 90 days of age and fixed in 4% paraformaldehyde for 24 h. After rinsing, gradient alcohol dehydration, xylene clearing, and paraffin embedding, the tissues were sectioned and stained with hematoxylin-eosin (HE). After mounting, villus height and crypt depth were observed and recorded under a microscope, and the ratio of villus height to crypt depth (V/C) was calculated for each intestinal segment.

### 2.8. Analysis of Intestinal Flora Structure

Total genomic DNA was extracted from jejunal chyme using the OMEGA Soil DNA Kit (Omega Bio-tek, Norcross, GA, USA). DNA size was verified via 0.8% agarose gel electrophoresis, and quantification was performed using a NanoDrop spectrophotometer (Thermo Fisher Scientific, Waltham, MA, USA). The V3-V4 hypervariable regions of the bacterial 16S rRNA gene were amplified using primers 338F (5′-barcode+ACTCCTACGGGAGGCAGCA-3′) and 806R (5′-GGACTACHVGGGTWTCTAAT-3′).

PCR was performed in a 25 μL reaction system using NEB Q5 High-Fidelity DNA Polymerase (New England Biolabs, Ipswich, MA, USA). The cycling conditions were: initial denaturation at 98 °C for 5 min; 25 cycles of 98 °C for 30 s, 53 °C for 30 s, and 72 °C for 45 s; and a final extension at 72 °C for 5 min. Amplicons were validated by 2% agarose gel electrophoresis, purified, quantified, and pooled in equimolar amounts. Sequencing libraries were constructed using the Illumina TruSeq Nano DNA LT Library Prep Kit (Illumina Inc., San Diego, CA, USA). High-throughput sequencing was performed on an Illumina platform, and the resulting raw data were analyzed using QIIME2 2022.11 software.

### 2.9. Transcriptome Profiling of the Jejunum

Total RNA was extracted from jejunal mucosal samples of 90-day-old geese using Trizol (Thermo Fisher Scientific, Waltham, MA, USA) method, and residual genomic DNA was digested with DNase I (Thermo Fisher Scientific, Waltham, MA, USA). The RNA purity was determined via a NanoDrop spectrophotometer (A260/A280 ratio > 2.0 for all samples), and its integrity was assessed using an Agilent Bioanalyzer (Agilent Technologies Inc., Santa Clara, CA, USA) (RIN values > 7.0). Eukaryotic mRNA was enriched with Oligo(dT) magnetic beads (Illumina Inc., San Diego, CA, USA), and double-stranded cDNA was synthesized after mRNA fragmentation. A sequencing library was constructed through end repair, adapter ligation, and PCR amplification, with the library quality verified by Qubit fluorometer (Thermo Fisher Scientific, Wilmington, DE, USA) and Agilent Bioanalyzer (Agilent Technologies Inc., Santa Clara, CA, USA).

Paired-end 150 bp sequencing was performed on the Illumina NovaSeq platform (Illumina Inc., San Diego, CA, USA). To ensure data quality, raw sequencing data were subjected to strict quality control using FastQC (v0.11.9) and filtered with Trimmomatic (v0.39) to remove adapters and low-quality reads (with a quality threshold of Q < 20). The resulting clean reads were aligned to the Anser cygnoides (goose) reference genome via Hisat2 (v2.2.1), achieving a valid mapping rate suitable for downstream analysis. Gene expression levels were quantified using HTSeq (v0.13.5). Differentially expressed genes (DEGs) between the CON and BA groups were screened with DESeq2 (v1.38.3) under the strict criteria of |log2FC| > 1 and FDR < 0.05. A total of 1253 DEGs were identified, comprising 317 upregulated and 936 downregulated genes in the BA group compared with the CON group. Functional annotation of DEGs was conducted against the GO and KEGG databases.

### 2.10. Validation of Transcriptomic Genes

Total RNA was extracted from jejunal mucosal samples via the Trizol method. Six differentially expressed genes (DEGs) were selected for qPCR validation based on the following predefined criteria: (1) biological relevance to intestinal barrier function, immune response, and antioxidant capacity (IL4I1, NOS2, SOCS2, MAL, IL13RA2); (2) representation of key pathways identified in KEGG enrichment analysis, including calcium signaling (NOXO1) and cell junction pathways (MAL); (3) genes exhibiting moderate to high fold changes (|log2FC| > 1 and FDR < 0.05) to ensure detectable expression differences; and (4) diverse functional categories to comprehensively validate the transcriptome results. β-actin was selected as the reference gene due to its stable expression across all samples (coefficient of variation < 5%).

To verify the RNA extraction quality, its integrity was detected by 1% agarose gel electrophoresis, and the concentration and purity were determined via ultraviolet (UV) spectrophotometry. Qualified RNA samples were then reverse-transcribed into cDNA using a reverse transcription kit. Finally, the expression levels of target genes were quantified on a real-time quantitative PCR instrument with the SYBR Green fluorescent quantitative system (Bio-Rad Laboratories, Inc., Hercules, CA, USA). Primer sequences are listed in Table 2.

The amplification efficiency for each primer pair, calculated via a standard curve, ranged from 90% to 110%. The relative expression levels of target genes were calculated using the 2^(−ΔΔCt)^ method, and a high consistency was observed between the qRT-PCR results and the RNA-Seq data, confirming the reliability of the transcriptomic profiling.

### 2.11. Data Analysis

Prior to statistical analysis, all data were tested for normality using the Shapiro–Wilk test and for homogeneity of variances using Levene’s test. Data satisfying these assumptions were analyzed using independent-samples *t*-test, while non-normally distributed data were analyzed using the Mann–Whitney U test. For multiple comparisons across different intestinal segments (duodenum, jejunum, ileum), Bonferroni correction was applied to control the family-wise error rate. For growth performance data (ADG, ADFI, FCR), the experimental unit was the pen (n = 6 pens per group), as feed intake was recorded at the pen level. Body weight was measured individually but analyzed as pen averages. For individual-level analyses, one goose was randomly selected from each of the 6 pens to avoid pen-level confounding; hence, the individual bird was the experimental unit (n = 6). Statistical analyses were performed using SPSS 25.0 (IBM Corp., Armonk, New York, NY, USA) and RStudio (version 4.2.1). Significance was declared at *p* < 0.05, and tendencies were noted at 0.05 ≤ *p* < 0.10. All results are presented as mean ± standard deviation (SD).

## 3. Results

### 3.1. Effects of Bacillus amyloliquefaciens on Growth Performance of Growing Geese

As shown in Table 3, dietary BA supplementation significantly influenced growth performance. There was no significant difference in initial body weight (at 50 d) between the groups (*p* > 0.05). However, at 70 and 90 days of age, the body weight of geese in the BA group was significantly higher than that of the CON group (*p* < 0.01). Average daily gain (ADG) was significantly increased in the BA group across all measured periods (50–70 d, 70–90 d, and 50–90 d) (*p* < 0.01). The ADFI of the BA group was significantly higher than that of the CON geese at 50~70 d and 70~90 d (*p* < 0.01), and there was no significant difference between BA and CON geese at 50~90 d (*p* > 0.05). Regarding FCR, the value for the BA group was significantly lower than that in the CON group (*p* < 0.01).

### 3.2. Effects of Bacillus amyloliquefaciens on Immune Performance of Growing Geese

The effects of dietary BA on serum immunity are presented in Table 4. Compared with the CON group, BA supplementation significantly increased serum IgA, IgG, and IgM levels (*p* < 0.01). Furthermore, BA significantly decreased serum IL-2, IL-1β, and TNF-α levels (*p* < 0.01), while significantly increasing IL-4 levels (*p* < 0.01).

### 3.3. Effects of Bacillus amyloliquefaciens on Intestinal Antioxidant Capacity of Growing Geese

The effects of BA on intestinal antioxidant capacity are shown in Table 5. In the duodenum, BA significantly increased CAT, SOD, GSH-Px, and T-AOC levels compared with the CON group (*p* < 0.01). In the jejunum, BA significantly increased GSH-Px (*p* < 0.05), CAT, and T-AOC (*p* < 0.01), while significantly decreasing MDA content (*p* < 0.01); jejunal SOD showed no significant change (*p* > 0.05). In the ileum, BA significantly increased CAT and T-AOC (*p* < 0.05), as well as GSH-Px (*p* < 0.01), and significantly decreased MDA (*p* < 0.05), with no significant change in SOD (*p* > 0.05).

### 3.4. Effects of Bacillus amyloliquefaciens on Intestinal Morphology of Growing Geese

**Figure 1:**
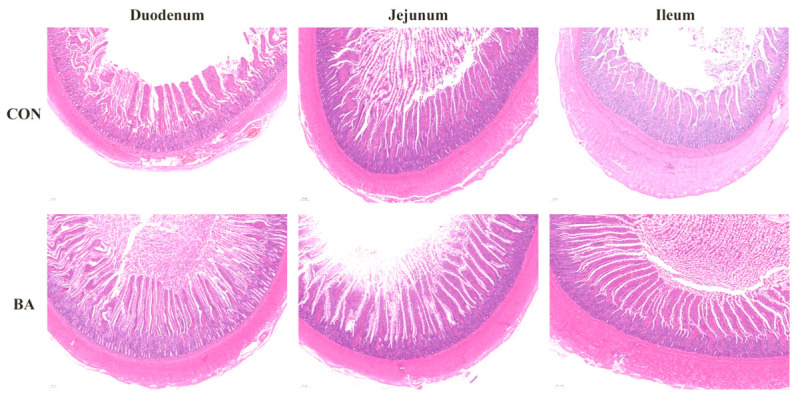
Effects of *Bacillus amyloliquefaciens* on intestinal morphological structure of growing geese. Note: Tissue sections were stained with hematoxylin-eosin (HE) (n = 6). CON, control group fed a basal diet; BA, *Bacillus amyloliquefaciens* group fed the basal diet supplemented with 1 × 10^8^ CFU/g BA. (Scale bar = 200 μm).

As shown in Figure 1 and Table 6, BA supplementation significantly increased villus height in the duodenum and jejunum (*p* < 0.01) but had no significant effect on the ileum (*p* > 0.05). Crypt depth in the duodenum was significantly elevated by BA (*p* < 0.05), whereas no significant differences were found in the jejunum or ileum (*p* > 0.05). Consequently, the V/C ratio in the jejunum was significantly increased in the BA group (*p* < 0.01), with no significant effects observed in the duodenum or ileum (*p* > 0.05).

### 3.5. Effects of Bacillus amyloliquefaciens on Intestinal Flora of Growing Geese

Table 7 presents the effects of dietary *Bacillus amyloliquefaciens* supplementation on the α-diversity indices of jejunal microbiota in geese. The richness indices, including ACE, Chao1, and Observed features, were not significantly different between the CON and BA groups (*p* > 0.05), despite numerical variations. Regarding community diversity, the Shannon index was significantly higher in the BA group than in the CON group (*p* < 0.01), while the Simpson index was significantly increased in the BA group (*p* < 0.05). The Pielou_e index tended to be greater in the BA group, but the difference did not reach statistical significance (*p* > 0.05). These results indicate that dietary *Bacillus amyloliquefaciens* supplementation may improve the evenness of the jejunal microbial community, although this effect was not statistically significant.

**Figure 2:**
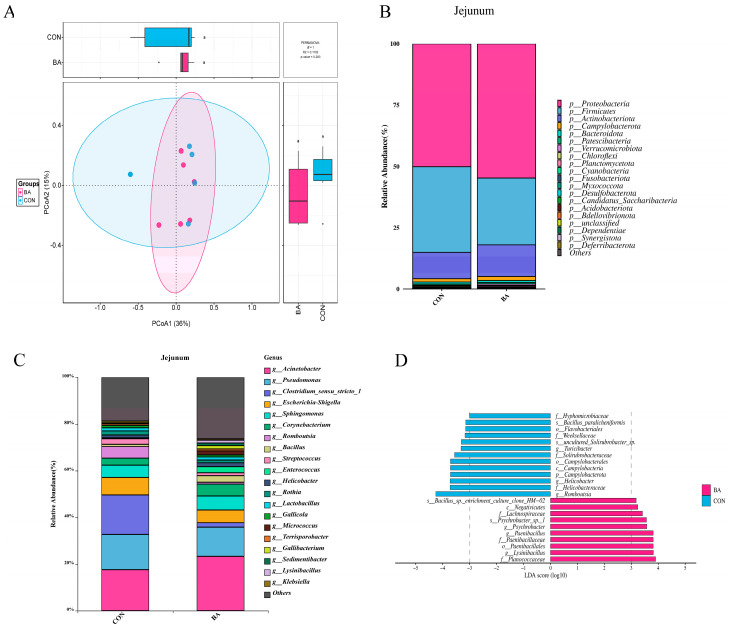
Effects of *Bacillus amyloliquefaciens* on jejunal flora of growing geese (n = 6). Note: CON, control group; BA, *B. amyloliquefaciens* group; (n = 6 individual geese per group, one per pen). (**A**) PCoA analysis of jejunal flora; (**B**) Species composition analysis of jejunal flora at the phylum level; (**C**) Species composition analysis of jejunal flora at the genus level; (**D**) LEfSe analysis of jejunal flora at the genus level. The letter a indicates no significant difference between CON and BA groups (*p* > 0.05).

To investigate the regulatory effect of *Bacillus amyloliquefaciens* on the composition of the jejunal microbial community, principal coordinate analysis (PCoA) was performed to resolve the overall differences in microbial community structure of this intestinal segment. As shown in Figure 2A, for the jejunal microbial community, PCoA1 and PCoA2 were the core dimensions driving community variations in the PCoA analysis. Although partial overlap was observed between the CON and BA samples, a distinguishable grouping trend was presented overall, indicating that significant differences existed in the composition of the jejunal microbial community between the two groups.

To further clarify the regulatory effect of *Bacillus amyloliquefaciens* on the jejunal microbial community, the species composition of the jejunal flora at the phylum and genus levels was analyzed. As shown in Figure 2B, in terms of community composition, the dominant taxon structure of the jejunal microbiota showed high consistency between the CON and BA groups. Proteobacteria was the absolutely dominant phylum in both groups, followed by Firmicutes. In addition to these two dominant phyla, the jejunal microbiota of both groups also contained minor taxa such as Actinobacteriota and Campylobacterota. At the genus level (Figure 2C), the community composition of the jejunal flora was relatively complex. In terms of dominant taxa, Acinetobacter was the core dominant genus in both groups, while a significant intergroup difference was found in its relative abundance: the relative abundance of Acinetobacter in the BA group was higher than that in the CON group, whereas the relative abundance of Pseudomonas in the CON group was higher than that in the BA group, showing an opposite variation trend in abundance. Furthermore, compared with the CON group, the BA group increased the abundance of Bacillus in the jejunum, which indicated the successful colonization of *Bacillus amyloliquefaciens*.

To identify the differential microbial taxa at the genus level between the CON and BA groups, linear discriminant analysis effect size (LEfSe) was conducted to screen the biomarker species between the two groups. As shown in Figure 2D, CON was mainly enriched in Campylobacterota, Helicobacter and Romboutsia, while the *Bacillus amyloliquefaciens* group showed significant enrichment of Psychrobacter, Paenibacillus, Lysinibacillus and Lachnospiraceae.

While the Shannon and Simpson indices indicated increased community diversity in the BA group, it is important to note that richness indices (ACE, Chao1, and Observed_features) did not differ significantly between groups. This discrepancy suggests that BA supplementation primarily enhanced the evenness of microbial distribution rather than increasing the total number of taxa. The biological significance of increased Shannon and Simpson indices without corresponding changes in richness remains to be fully elucidated. Furthermore, although PCoA revealed a discernible grouping trend between the CON and BA groups, partial overlap was observed, indicating that inter-individual variation in microbial composition was substantial and that BA-induced shifts, while statistically significant, were moderate in magnitude. Therefore, the interpretation of increased microbial diversity as universally ‘beneficial’ should be made with caution, as the functional consequences of these community shifts require further validation through metagenomic or metabolomic approaches.

### 3.6. Correlation Analysis of Intestinal Flora with Immune Performance and Antioxidant Function

**Figure 3:**
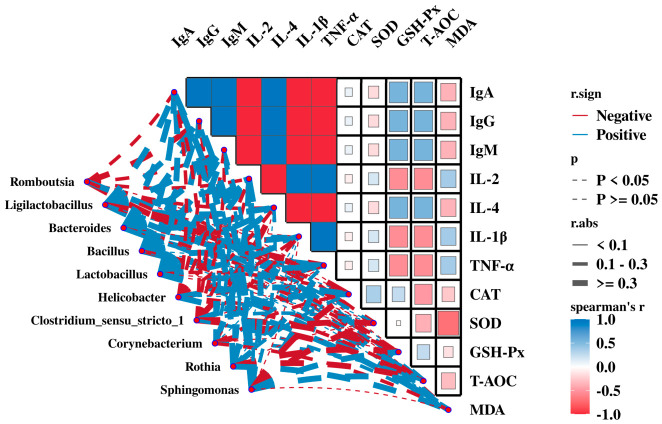
Correlation analysis between jejunal genera and antioxidant as well as immune indices. Note: The heatmap was generated using Spearman correlation analysis (n = 6). CON, control group fed a basal diet; BA, *Bacillus amyloliquefaciens* group fed the basal diet supplemented with 1 × 10^8^ CFU/g BA. CAT, catalase; SOD, superoxide dismutase; GSH-Px, glutathione peroxidase; T-AOC, total antioxidant capacity; MDA, malondialdehyde; IgA, immunoglobulin A; IgG, immunoglobulin G; IgM, immunoglobulin M; IL, interleukin; TNF-α, tumor necrosis factor-α.

Spearman correlation analysis was conducted to evaluate the relationships between differential microbial genera and host immune or antioxidant parameters (Figure 3; detailed data in Table S1). Among beneficial genera, Ligilactobacillus was significantly positively correlated with IgA and IgG (*p* < 0.05) and positively correlated with IgM and IL-4 (*p* > 0.05). Bacteroides exhibited significant positive correlations with IgA, IgG, IgM, and IL-4 (*p* < 0.05). Bacillus abundance was significantly positively correlated with IgA, IgG, IgM, and IL-4 (*p* < 0.05), and positively correlated with CAT and SOD (*p* > 0.05). Lactobacillus showed significant positive correlations with IgA, IgG, IgM, and IL-4 (*p* < 0.05), and positive correlations with T-AOC and MDA (*p* > 0.05).

Conversely, potential pathogenic genera, including Romboutsia, Helicobacter, and Clostridium_sensu_stricto_1, were mostly negatively correlated with immunoglobulins and anti-inflammatory cytokines (*p* > 0.05).

### 3.7. Effects of Bacillus amyloliquefaciens on Intestinal Transcriptome of Growing Geese

**Figure 4:**
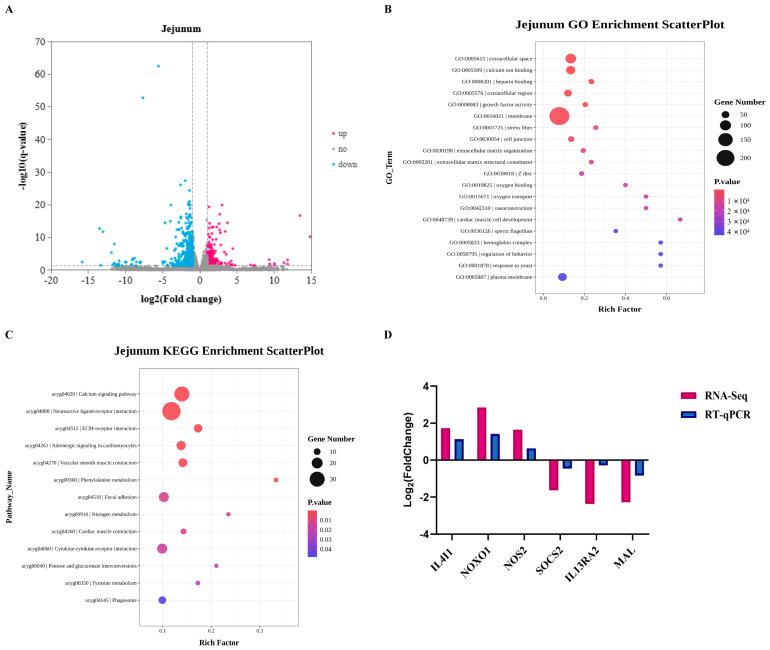
Jejunum transcriptome results (n = 6). Note: CON, control group fed a basal diet; BA, *Bacillus amyloliquefaciens* group fed the basal diet supplemented with 1 × 10^8^ CFU/g BA. (**A**) Jejunum gene expression volcano map; (**B**) Jejunum GO enrichment analysis; (**C**) KEGG enrichment analysis of jejunum; (**D**) Validation of transcriptional gene expression level.

Transcriptome sequencing analysis was performed on jejunal tissues, and differentially expressed genes (DEGs) were screened with the thresholds of |log_2_ (Fold Change)| ≥ 1 and *q* < 0.05. As shown in Figure 4A, *Bacillus amyloliquefaciens* upregulated 317 genes and downregulated 936 genes in the jejunum of growing geese.

### 3.8. GO Enrichment Analysis of Intestinal Tissues Regulated by Bacillus amyloliquefaciens

To systematically elaborate on the tissue-specific effects of dietary probiotic supplementation on the physiological functions of different intestinal segments in geese, GO enrichment analysis was performed on the differentially expressed genes (DEGs) identified in the jejunum. A total of 326 significantly enriched GO terms were obtained. To reduce the interference of data redundancy and background noise on the analysis results, the top 5–8 significantly enriched terms were selected from each of the three subcategories of GO functional annotation (biological process [BP], molecular function [MF], and cellular component [CC]) for subsequent detailed analysis and visualization.

As shown in Figure 4B, at the CC level, the core enrichment directions of jejunal DEGs were focused on terms related to membrane structure and extracellular microenvironment, including extracellular space, membrane, extracellular region, cell junction, and plasma membrane, all of which were highly ranked enriched terms. Among these, the extracellular space exhibited the highest enrichment significance (*p* ≤ 10^−4^), the membrane was associated with the largest number of DEGs, and the enrichment factor of cell junction exceeded 0.2. These results suggest that *Bacillus amyloliquefaciens* could affect the transmembrane transport of substances, the integrity of barrier structure, and the interaction with the external microenvironment by regulating the membrane structure of jejunal epithelial cells and the components of the extracellular microenvironment.

At the MF level, calcium ion binding and heparin binding were the core enriched terms. Among them, calcium ion binding ranked first in the MF category both in terms of enrichment significance (*p* ≤ 10^−4^) and the number of DEGs, reflecting its core regulatory role in jejunal ion signal transduction. Meanwhile, the jejunum specifically enriched terms of growth factor activity and oxygen binding: growth factor activity is involved in mediating the proliferation and damage repair signals of jejunal epithelial cells, while oxygen binding supports the balance of oxygen supply and demand in intestinal tissues. These two terms jointly participate in maintaining the nutritional absorption and structural homeostasis of the jejunum.

At the BP level, jejunal DEGs were mainly enriched in terms related to local microenvironment regulation and response to external stimuli. The enrichment of vasoconstriction suggested that *Bacillus amyloliquefaciens* could optimize the distribution of nutrients and the clearance of metabolic wastes by regulating the local hemodynamics of the jejunum. The enrichment of response to yeast reflected its potential role in resisting the abnormal proliferation of intestinal commensal microorganisms and maintaining the steady state of jejunal flora.

Collectively, the GO enrichment analysis revealed that *Bacillus amyloliquefaciens* may regulate the jejunal physiological function of growing geese by inducing differential expression of genes involved in membrane structure modulation, extracellular microenvironment regulation, ion signal transduction, epithelial cell repair, and flora homeostasis. These findings are consistent with the results of intestinal morphology, immune performance, antioxidant capacity, and intestinal flora analysis and lay a molecular foundation for further clarifying the tissue-specific regulatory mechanism of *Bacillus amyloliquefaciens* on intestinal health in geese.

### 3.9. KEGG Enrichment Analysis of Intestinal Tissue by Bacillus amyloliquefaciens

As shown in Figure 4C, the integrated KEGG pathway enrichment analysis of the jejunum revealed that the highly significantly enriched pathways (*p* ≤ 0.01) of jejunal DEGs included the calcium signaling pathway, neuroactive ligand-receptor interaction, and extracellular matrix (ECM)–receptor interaction. These pathways exhibited high enrichment factors and a large number of DEGs, serving as the core pathways underlying the differential functions of the jejunum. In contrast, pathways such as phenylalanine metabolism and phagosome showed moderate to low significant enrichment (*p* ≥ 0.02), with relatively low proportions and numbers of DEGs. The above highly significantly enriched pathways are mainly involved in functional directions such as signal transduction and cell–matrix interaction, suggesting that dietary supplementation with *Bacillus amyloliquefaciens* can regulate these core pathways to affect the signal transmission and cell–cell interaction processes of the jejunum, thereby participating in the regulation of jejunal physiological functions.

To verify the reliability of the RNA-Seq data, six DEGs were selected for qRT-PCR verification to confirm their expression trends in the jejunum, which were consistent with those obtained from high-throughput sequencing. These results confirm the reliability of the transcriptome data.

## 4. Discussion

Against the background of the antibiotic ban, probiotic supplements have developed rapidly owing to their ability to regulate intestinal flora and have become an important and ideal strategy for maintaining intestinal health in intensive poultry production. *Bacillus amyloliquefaciens* is an endospore-forming, Gram-positive, facultatively anaerobic bacterium. Previous studies have shown that *Bacillus amyloliquefaciens*, as a feed additive, can improve feed conversion efficiency, enhance animal growth performance, and strengthen host immune function [9,10,11].

Growth performance is a key indicator of overall health and nutritional status, directly impacting the economic viability and productivity of the poultry industry. Ahmat et al. [12] supplemented broiler diets with 5 × 10^8^ CFU/kg *Bacillus amyloliquefaciens* LFB112 and significantly increased body weight and average daily gain, while reducing feed conversion ratio, which was consistent with the findings of Hong et al. [13]. In the present study, dietary supplementation with *Bacillus amyloliquefaciens* significantly increased final body weight and average daily gain, and decreased feed conversion ratio in geese. It only significantly promoted average daily feed intake during 70–90 d, with no significant difference in overall feed intake, suggesting that the improvement in growth performance was mainly attributed to optimized feed efficiency rather than a general increase in feed intake.

*Bacillus amyloliquefaciens* is known to secrete a range of digestive enzymes, including amylase, protease, cellulase, and lipase. These enzymes specifically catalyze the degradation of carbohydrates, proteins, lipids, and other nutrients in feed, thereby facilitating their absorption and improving overall nutrient digestibility [7,8]. The improved feed conversion ratio (FCR) and increased body weight gain observed in the *Bacillus amyloliquefaciens*-supplemented group in this study may be attributed to the enhanced nutrient digestion and absorption mediated by these enzymes, which constitutes a key pathway underlying the positive regulatory effect of this probiotic on goose growth performance.

In poultry farming, antioxidant capacity is critical for maintaining health and production performance and is also a core indicator for evaluating resistance to endogenous oxidative damage. When the antioxidant system is imbalanced and free radical scavenging capacity is weakened, the accumulation of free radicals triggers oxidative stress, disrupts cell membrane integrity, impairs membrane transport and signal transduction, interferes with key cellular enzymatic reactions, and further inhibits the synthesis and secretion of growth hormone and growth signal transduction, thereby significantly affecting poultry growth, development, and production performance [14]. The antioxidant defense system plays a central role in maintaining host antioxidant capacity, in which key antioxidant enzymes such as catalase (CAT), glutathione peroxidase (GSH-Px), and superoxide dismutase (SOD) exert core effects [15].

Numerous studies have confirmed that Bacillus species can effectively improve antioxidant capacity and alleviate oxidative damage in poultry. Xu et al. [16] supplemented broiler diets with B. subtilis and B. licheniformis preparations, respectively, and found that serum GSH-Px, SOD, and CAT activities were significantly increased, while malondialdehyde (MDA) content was significantly decreased, indicating that these two Bacillus strains enhanced antioxidant function in broilers. Similar antioxidant effects were observed by Zhang et al. [17] when supplementing broilers with B. coagulans. Wang et al. [18] reported that *Bacillus amyloliquefaciens* SCO6 significantly increased hepatic T-AOC and T-SOD activities in broilers.

The results of the present study showed that dietary supplementation with *Bacillus amyloliquefaciens* significantly elevated CAT, GSH-Px, and T-AOC levels in the duodenum, jejunum, and ileum of geese, and markedly reduced MDA content in the jejunum, effectively improving host antioxidant capacity. These findings are consistent with previous studies demonstrating that Bacillus strains can enhance antioxidant capacity in animals.

Host health relies heavily on the immune system. Immunoglobulins are core executors of humoral immunity and play an important role in defending against pathogenic infections. Existing studies have shown that adding 0.01% *Bacillus amyloliquefaciens* to layer diets significantly reduced serum IL-1β and TNF-α contents and increased IL-4 content [19]. Wang et al. [18] found that *Bacillus amyloliquefaciens* significantly increased IL-10 concentration and decreased IL-6 and TNF-α concentrations in the ileum of broilers. The results of the present experiment indicated that dietary *Bacillus amyloliquefaciens* significantly decreased the concentrations of IL-2, IL-1β, and TNF-α, and significantly increased IL-4 concentration in geese, demonstrating that *Bacillus amyloliquefaciens* suppressed inflammatory responses and enhanced anti-inflammatory capacity.

The slender finger-like protrusions on the surface of the small intestinal mucosa are intestinal villi, and crypts are invaginations in the mucosal layer corresponding to villi. Together, they constitute the highly efficient functional unit for small intestinal nutrient absorption and play a key role in maintaining mucosal structural stability, strengthening the intestinal physical barrier, resisting pathogen invasion, and supporting efficient nutrient digestion and absorption as well as normal physiological activities [20,21]. Many studies have confirmed that *Bacillus amyloliquefaciens* can improve intestinal morphology in broilers by significantly increasing villus height, crypt depth, and villus-height-to-crypt-depth ratio [22,23,24].

The results of the present study show that dietary supplementation with *Bacillus amyloliquefaciens* significantly increased villus height and crypt depth in the duodenum and significantly affected villus height and the V/C ratio in the jejunum of geese, effectively improving intestinal morphology. As the initial segment of the small intestine, the duodenum connects the gizzard and jejunum and serves as the core site for chemical digestion. Increased villus height expands the intestinal absorption surface area, which is generally associated with enhanced nutrient absorption capacity [20,21]. The interpretation of crypt depth changes is more complex: while deeper crypts may reflect increased epithelial cell proliferation and renewal capacity, which could support villus growth, some studies suggest that excessively deep crypts may indicate ongoing mucosal damage or inflammatory responses that require increased regenerative activity [25]. In the present study, BA supplementation increased crypt depth in the duodenum but not in the jejunum or ileum, and the V/C ratio (a more integrative indicator of mucosal health) was significantly improved only in the jejunum. Therefore, the functional significance of the observed crypt depth increase should be interpreted cautiously, considering the segment-specific responses and the lack of direct proliferation markers (e.g., Ki-67, PCNA) or apoptotic indices.

The digestive and absorptive functions of animals are closely related to intestinal flora, and the homeostasis of intestinal flora is a core hub regulating host immune function, maintaining inflammatory balance, and modulating oxidative stress. The results of this experiment show that dietary supplementation with *Bacillus amyloliquefaciens* significantly altered the composition and structure of intestinal flora in young geese, and these microbial changes were closely correlated with improved growth performance and optimized physiological functions. *Bacillus amyloliquefaciens* affected the diversity and overall structure of intestinal flora. In the jejunum, the Shannon and Simpson indices were significantly increased in the *Bacillus amyloliquefaciens* group, which was consistent with the findings of Zhang et al. [26] that *Bacillus amyloliquefaciens* may enhance intestinal flora diversity in animals.

The observation of increased Shannon and Simpson indices without significant changes in ACE, Chao1, or Observed_features suggests that *B. amyloliquefaciens* primarily modulated the relative abundance of existing taxa rather than introducing novel microbial species. This pattern of ‘evenness-driven’ diversity increase differs from ‘richness-driven’ diversity, and their ecological and functional implications may differ. While higher evenness is generally associated with enhanced ecosystem stability and resistance to perturbation in macroecological systems, its direct relevance to intestinal health in poultry requires further investigation. The partial overlap observed in PCoA also underscores the importance of considering effect sizes alongside statistical significance when interpreting microbiome data. Future studies incorporating larger sample sizes and functional metagenomic analyses are needed to definitively link these community structural changes to host physiological outcomes.

Notably, although the phylum Proteobacteria was found to be significantly higher in the BA group, this expansion should not be simply interpreted as a typical marker of gut dysbiosis. A closer examination at the genus level revealed that the increase in Proteobacteria was primarily driven by the enrichment of specific genera such as Acinetobacter, Psychrobacter, Paenibacillus, and Lysinibacillus. Among these, Paenibacillus and Lysinibacillus are well-documented as beneficial bacteria capable of producing antimicrobial peptides and promoting nutrient digestion. Crucially, the relative abundances of potentially pathogenic genera within the same phylum, such as Pseudomonas and Escherichia–Shigella, were significantly decreased in the BA group. Therefore, the increase in Proteobacteria observed in this study represents a beneficial remodeling of the microbial community—where commensal or beneficial taxa outcompeted opportunistic pathogens—rather than a detrimental dysbiotic shift, which is consistent with the improved growth and immune parameters observed in the geese.

At the genus level, the relative abundances of opportunistic pathogens such as Pseudomonas and Escherichia–Shigella were significantly decreased in the jejunum of the *Bacillus amyloliquefaciens* group. The core mechanisms may involve two aspects: first, *Bacillus amyloliquefaciens* directly inhibits the proliferation of opportunistic pathogens by competing for nutrients and ecological niches and secreting antibacterial metabolites such as bacteriocins; second, the potential production of bacteriocins and metabolites may create an unfavorable microenvironment for these pathogens, ultimately synergistically reshaping a healthy intestinal microbial community structure.

The remodeling of the gut microbiota induced by *Bacillus amyloliquefaciens* may represent a key driver in regulating immune function, inflammatory responses, and antioxidant capacity within the jejunum of Zi geese. This regulatory mechanism is closely linked to the metabolic characteristics of both beneficial and pathogenic bacteria, as well as their interplay with the host. Spearman correlation analysis revealed that beneficial genera, including Ligilactobacillus, Bacteroides, Bacillus, and Lactobacillus, were significantly and positively correlated with serum IgA, IgG, and IgM levels. In contrast, Psychrobacter and Lysinibacillus—which were markedly enriched in the *B. amyloliquefaciens*-supplemented group—showed no significant correlations with immune or antioxidant indices. These findings suggest that the immunomodulatory effects of probiotic supplementation are likely mediated primarily through the suppression of potentially harmful bacteria such as Romboutsia.

It is speculated that these beneficial bacteria can bind to surface receptors of intestinal mucosal immune cells, trigger host humoral immune responses, promote immunoglobulin synthesis and secretion, and thereby strengthen the immune defense function of the intestinal mucosal barrier [27,28]. Meanwhile, it is plausible that bacterial metabolism, including that of beneficial genera enriched by BA supplementation, may produce SCFAs and other bioactive metabolites that influence host physiology. However, as SCFA concentrations were not directly measured in the present study, this mechanism remains speculative and requires direct experimental validation [29].

In addition, the positive correlations between beneficial genera and anti-inflammatory cytokines suggest potential immunomodulatory effects of BA-induced microbiota remodeling. While previous studies in other species have implicated the NF-κB signaling pathway in probiotic-mediated anti-inflammatory effects, the present study did not directly measure NF-κB activity, SCFA concentrations, or intestinal pH. Therefore, the specific molecular mechanisms underlying these correlations cannot be conclusively determined from our data and warrant further investigation. SCFAs can block the initiation of excessive inflammatory responses, regulate the proliferation and differentiation of intestinal epithelial cells, enhance the structural integrity of the intestinal mucosal barrier, and reduce the occurrence of inflammation at the source [30,31].

At the antioxidant level, beneficial genera were positively correlated with antioxidant indices, including CAT, SOD, and T-AOC (*p* > 0.05). Based on previous literature, it is speculated that probiotics and their metabolites may upregulate the transcription of antioxidant enzyme-encoding genes via specific antioxidant signaling pathways (e.g., Nrf2/ARE). However, the exact molecular pathways involved in the present study require further investigation [32,33,34].

The present study also found that the relative abundances of harmful jejunal genera, including Romboutsia, Helicobacter, and Clostridium, were negatively correlated with immunoglobulins (IgA, IgG, IgM), IL-4, GSH-Px, and T-AOC. The core harm of such harmful bacteria lies in the disruption of host physiological functions via their metabolic characteristics. Helicobacter, Escherichia–Shigella and other genera can secrete cytotoxins or endotoxins, damage intestinal mucosal epithelial integrity, and suppress immune cell activation and immunoglobulin synthesis [34,35]. Meanwhile, excessive proliferation of Helicobacter induces oxidative stress, depletes endogenous antioxidant enzymes, reduces activities such as GSH-Px, and aggravates intestinal oxidative damage [36].

Transcriptomic analysis revealed enrichment of DEGs in pathways related to cell junctions and ECM–receptor interactions, suggesting that BA may influence the molecular machinery underlying intestinal barrier integrity. However, it is important to acknowledge that the present study did not directly measure tight junction protein expression (e.g., occludin, claudin, ZO-1), transepithelial electrical resistance (TEER), or intestinal permeability (e.g., FITC-dextran assay). Therefore, while our morphological and transcriptomic data are consistent with improved barrier function, direct evidence for enhanced intestinal barrier integrity is lacking, and this interpretation should be considered preliminary.

GO enrichment analysis of cellular components revealed that DEGs were significantly enriched in terms including “membrane”, “cell junction”, and “extracellular region”, directly indicating that the action targets of *Bacillus amyloliquefaciens* are concentrated on the intestinal epithelial interface structure. Regulation of cell junctions is fundamental for maintaining intestinal selective permeability. Combined with the significant enrichment of the “ECM–receptor interaction” pathway in KEGG analysis, it suggests that *Bacillus amyloliquefaciens* may be associated with the molecular machinery underlying intestinal physical barrier maintenance. While these transcriptomic shifts provide a potential molecular explanation for the improved intestinal morphology observed in this experiment, they require direct validation through protein-level assessments in future studies.

In summary, *Bacillus amyloliquefaciens* may take the calcium signaling pathway as a core regulatory hub, simultaneously modulating cell–extracellular matrix (ECM) interactions and cell junction assembly. These transcriptomic shifts, alongside improved morphology and microbiota remodeling, suggest a potential role for BA in supporting jejunal barrier function and internal environmental homeostasis, although direct physiological validation is required.

Several limitations of the present study should be acknowledged to provide a balanced view of the findings. First, the absence of an antibiotic-positive control group limits the direct comparison of BA efficacy with conventional growth promoters, which would be valuable for practical application. Second, the evaluation of only one BA dose, while based on preliminary data, precludes the establishment of a dose–response relationship. Third, the relatively small sample size (n = 6) for microbiome and transcriptome analyses, although consistent with previous poultry studies, may limit the detection of subtle microbial shifts and increase the risk of false negatives. Fourth, the 40-day experimental period captures the growing phase but does not address long-term effects or persistence of BA benefits. Fifth, while our transcriptomic and microbiome analyses provide correlative insights, mechanistic validation through pathway-specific inhibitors, gene silencing, or germ-free models is needed to establish causality. Sixth, the absence of direct barrier function measurements (tight junction protein immunohistochemistry or Western blotting, TEER, or permeability assays) represents a significant limitation in definitively concluding that BA enhances intestinal barrier function; future studies should incorporate these complementary approaches. Seventh, the qPCR validation of transcriptomic data should be interpreted with caution. In silico analysis indicated potential off-target binding for several primer pairs, and the lack of empirical melt curve data precludes the definitive confirmation of amplification specificity. Therefore, the qPCR results serve as a supportive rather than conclusive validation. Finally, we must acknowledge a significant anatomical constraint regarding our microbiota analysis. This study exclusively focused on the jejunal microbiome; however, it is well established that microbial fermentation and short-chain fatty acid (SCFA) generation in geese occur predominantly in the hindgut, particularly the cecum. Consequently, the present analysis does not capture the tremendous microbial dynamics in the hindgut. Future studies are highly recommended to adopt a multi-segmental approach (jejunum, ileum, and cecum) and integrate SCFA metabolomics to provide a more comprehensive understanding of the spatial-specific mechanisms of probiotics in geese.

In comparison with existing literature, some studies have reported inconsistent effects of Bacillus probiotics on poultry growth performance. For example, Krueger [37] found no significant improvement in broiler growth with B. subtilis supplementation at similar doses, possibly due to differences in strain virulence, basal diet composition, or environmental stress levels. The discrepancy highlights the strain-specific and context-dependent nature of probiotic effects, emphasizing the need for standardized reporting of strain characteristics, experimental conditions, and host factors in future studies.

## 5. Conclusions

The present study confirmed that dietary supplementation with *Bacillus amyloliquefaciens* during the growing period could effectively improve the growth performance, immune function, and intestinal health of Zi geese. Specifically, this probiotic significantly increased the average daily gain and feed conversion efficiency, enhanced the immune function and antioxidant capacity, and improved the intestinal morphology and the homeostasis of intestinal microbial structure. Transcriptome analysis further revealed that its regulatory effects were closely related to the expression regulation of intestinal barrier function genes associated with the calcium signaling pathway and cell junctions. In summary, dietary supplementation with 1 × 10^8^ CFU/g *Bacillus amyloliquefaciens* for growing Zi geese can be used as a potential green feed additive to promote the healthy breeding of geese, taking into account production benefits and the sustainable development of the industry.

## Figures and Tables

**Table 1:** Base diet composition and nutritional level.

Item	Content
Ingredients %	
Corn	61.50
Soybean meal (CP43%)	25.00
Rice bran	7.00
Wheat bran	3.00
Stone powder	1.00
DL-Met (98%)	0.10
CaHPO4	1.10
NaCl	0.30
Premix ^(1)^	1.00
Total	100.00
Nutrient levels	
Metabolizable energy, MJ/kg ^(2)^	10.92
Crude protein	16.02
Crude fiber	5.60
Calcium	0.72
Phosphorus	0.54
Lysine	0.80
Methionine	0.33

Note: (1) Premix for each kilogram of concentrate: zinc 60 mg, copper 7 mg, manganese 80 mg, iron 50 mg, iodine 0.42 mg, selenium 0.3 mg, vitamin A 8000 IU, vitamin E 20 mg, vitamin D3 700 IU, vitamin K 2 mg, niacin 60 mg, pantothenic acid 15 mg, biotin 0.1 mg. (2) Metabolic energy was calculated according to the Chinese Feed Composition and Nutritional Value Table (34th edition, 2023), and the rest were measured values.

**Table 2:** Sequences of primers used for quantitative real-time PCR.

Gene	Primer Sequence (5′-3′)	Accession NO.	Size, bp	Annealing T, °C
IL4I1	F:CACGCCTGGATCGATACCTC R:AAGGTTGGAGCGTCTCACAG	XM_048055390.2	146	60
NOXO1	F:TGACTCAGACACAGCTGCTG R:CTTTGTGACTGTGCTGCACC	XM_013174976.3	88	60
NOS2	F:TCCATGGCCTCATTGCACAT R:CAGTAGCCACCTTCAGTGCA	XM_013193495.3	85	60
SOCS2	F:GTCCCGACTGAAGCAGTTCA R:GATGGAGCCGTCGTGTAGAG	XM_013182549.2	138	60
IL13RA2	F:AAGGGCAACACCTCACACAA R:CCCCACTTCAGCTTTGCAAC	XM_048080701.2	170	60
MAL	F:GAGGGAAGGAGGCTGCATTT R:TGGAAGTTGCCGAGGACATC	XM_013196143.3	87	60
β-actin	F:CCCAGCCATGTATGTAGCCATCC R:AACACCATCACCAGAGTCCATCAC	XM_013174886.1	91	60

F: forward primer; R: reverse primer.

**Table 3:** Effects of dietary *Bacillus amyloliquefaciens* on growth performance of geese.

Item	Group		*p*-Value
	CON	BA	
BW, g			
50 d	1247.6 ± 1.96	1247.2 ± 1.74	0.883
70 d	2663.8 ± 15.08	2754 ± 10.95	<0.01
90 d	3510.80 ± 25.17	3899.4 ± 8.45	<0.01
ADG, g			
50~70 d	70.81 ± 0.72	75.34 ± 0.59	<0.01
70~90 d	42.35 ± 1.51	57.27 ± 0.70	<0.01
50~90 d	56.58 ± 0.66	66.31 ± 0.22	<0.01
ADFI, g			
50~70 d	256.95 ± 2.73	274.17 ± 1.87	<0.01
70~90 d	298.20 ± 0.13	299.01 ± 0.04	<0.01
50~90 d	259.58 ± 4.84	265.05 ± 3.06	0.367
FCR			
50~90 d	4.59 ± 0.06	4.00 ± 0.05	<0.01

Note: Data are presented as mean ± standard deviation (SD) (n = 6 pens per group, 15 geese per pen). CON, control group fed a basal diet; BA, *B. amyloliquefaciens* group fed the basal diet supplemented with 1 × 10^8^ CFU/g BA. BW, body weight; ADG, average daily gain; ADFI, average daily feed intake; FCR, feed conversion ratio. Feed intake was recorded at the pen level, and FCR was calculated as total pen feed intake divided by total pen weight gain.

**Table 4:** Effects of dietary *Bacillus amyloliquefaciens* on serum immune indexes of geese.

Item	Group		*p*-Value
	CON	BA	
IgA, g/L	9.74 ± 0.29	13.42 ± 0.41	<0.01
IgG, g/L	28.32 ± 0.47	30.03 ± 0.19	<0.01
IgM, g/L	9.22 ± 0.27	10.29 ± 0.18	<0.01
IL-2, pg/mL	366.96 ± 7.74	325.36 ± 4.41	<0.01
IL-4, pg/mL	73.33 ± 2.11	83.37 ± 1.13	<0.01
IL-1β, pg/mL	134.17 ± 1.88	108.35 ± 1.96	<0.01
TNF-α, pg/mL	407.74 ± 8.55	359.10 ± 3.56	<0.01

Note: Data are presented as mean ± SD (n = 6). CON, control group fed a basal diet; BA, *Bacillus amyloliquefaciens* group fed the basal diet supplemented with 1 × 10^8^ CFU/g BA. IgA, immunoglobulin A; IgG, immunoglobulin G; IgM, immunoglobulin M; IL-2, interleukin-2; IL-4, interleukin-4; IL-1β, interleukin-1β; TNF-α, tumor necrosis factor-α.

**Table 5:** Effects of dietary *Bacillus amyloliquefaciens* on intestinal antioxidant capacity of geese.

Item	Tissue	Group		*p*-Value
		CON	BA	
CAT, U/g prot	duodenum	3.04 ± 0.06	3.86 ± 0.17	<0.01
	jejunum	5.32 ± 0.07	7.08 ± 0.03	<0.01
	ileum	5.53 ± 0.07	5.79 ± 0.07	0.019
SOD, U/g prot	duodenum	0.93 ± 0.02	1.07 ± 0.02	<0.01
	jejunum	0.95 ± 0.02	0.91 ± 0.02	0.146
	ileum	0.97 ± 0.03	1.05 ± 0.03	0.054
GSH-Px, U/g prot	duodenum	3.07 ± 0.05	3.82 ± 0.09	<0.01
	jejunum	5.67 ± 0.11	6.16 ± 0.11	0.012
	ileum	4.82 ± 0.06	5.90 ± 0.06	<0.01
T-AOC, U/g prot	duodenum	0.0098 ± 0.00028	0.0146 ± 0.00053	<0.01
	jejunum	0.0095 ± 0.00013	0.0105 ± 0.0001	<0.01
	ileum	0.01 ± 0.00025	0.0109 ± 0.0001	0.01
MDA, U/g prot	duodenum	0.22 ± 0.02	0.20 ± 0.01	0.258
	jejunum	0.13 ± 0.01	0.10 ± 0.002	<0.01
	ileum	0.15 ± 0.009	0.11 ± 0.005	0.011

Note: Data are presented as mean ± SD (n = 6). CON, control group fed a basal diet; BA, *Bacillus amyloliquefaciens* group fed the basal diet supplemented with 1 × 10^8^ CFU/g BA. CAT, catalase; SOD, superoxide dismutase; GSH-Px, glutathione peroxidase; T-AOC, total antioxidant capacity; MDA, malondialdehyde.

**Table 6:** Effects of dietary *Bacillus amyloliquefaciens* on intestinal tissue morphology of geese.

Item	Tissue	Group		*p*-Value
		CON	BA	
Villus height, μm	duodenum	912.80 ± 12.04	1120.97 ± 11.33	<0.01
	jejunum	598.97 ± 30.57	1024.63 ± 22.15	<0.01
	ileum	640.53 ± 21.43	731.30 ± 53.20	0.189
Crypt depth, μm	duodenum	338.80 ± 8.44	398.27 ± 18.05	0.041
	jejunum	395.77 ± 20.03	405.47 ± 22.41	0.763
	ileum	428.60 ± 18.57	399.73 ± 11.27	0.255
V/C ratio	duodenum	2.70 ± 0.03	2.83 ± 0.15	0.448
	jejunum	1.52 ± 0.06	2.54 ± 0.09	<0.01
	ileum	1.50 ± 0.10	1.84 ± 0.18	0.17

Note: Data are presented as mean ± SD (n = 6). CON, control group fed a basal diet; BA, *Bacillus amyloliquefaciens* group fed the basal diet supplemented with 1 × 10^8^ CFU/g BA. V/C, villus height to crypt depth ratio.

**Table 7:** Effects of *Bacillus amyloliquefaciens* on Intestinal Flora of Growing Geese.

Item	Group		*p*-Value
	CON	BA	
ACE	269.29 ± 37.85	394.38 ± 60.58	0.118
Chao1	272.55 ± 38.24	393.09 ± 60.04	0.129
Shannon	5.07 ± 0.15	5.95 ± 0.19	<0.01
Simpson	0.93 ± 0.01	0.96 ± 0.01	0.031
Pielou_e	0.64 ± 0.02	0.71 ± 0.03	0.123
Observed_features	249.00 ± 32.91	358.60 ± 48.29	0.098

Note: Data are presented as mean ± SD (n = 6 individual geese per group, one goose randomly selected from each of the 6 pens). CON, control group fed a basal diet; BA, *B. amyloliquefaciens* group fed the basal diet supplemented with 1 × 10^8^ CFU/g BA. Samples were collected from individual birds and analyzed separately without pooling.

## Data Availability

The data presented in this study are available on request from the corresponding author.

## Supplementary Materials

The following supporting information can be downloaded at: https://www.mdpi.com/article/10.3390/ani16142243/s1, Table S1: Correlation analysis between genus-level jejunal microbiota and antioxidant and immune indices.
