# Supplementary material for: Effects of Bacillus amyloliquefaciens on Growth Performance, Immune Performance, Antioxidant Capacity, Jejunal Microbiota and Transcriptome of Zi Geese
Source: Animals (Basel). 2026 Jul 20;16(14):2243. doi: 10.3390/ani16142243 (PMC13404800; doi:10.3390/ani16142243)
Supplement: Supplementary file 1 [file animals-16-02243-s001.zip › animals-4436348-supplementary.pdf]

Table S1 Correlation analysis between genus-level jejunal microbiota and antioxidant and immune indices

spec	env	r	p	rd	r.sign	pd
Romboutsia	IgA	-0.23939602	0.598611111	0.1 - 0.3	Negative	P >= 0.05
Romboutsia	IgG	-0.302669571	0.913888889	>= 0.3	Negative	P >= 0.05
Romboutsia	IgM	-0.240011742	0.665277778	0.1 - 0.3	Negative	P >= 0.05
Romboutsia	IL-2	-0.272788947	0.75	0.1 - 0.3	Negative	P >= 0.05
Romboutsia	IL-4	-0.231266918	0.706944444	0.1 - 0.3	Negative	P >= 0.05
Romboutsia	IL-1β	-0.071259969	0.4375	< 0.1	Negative	P >= 0.05
Romboutsia	TNF-α	-0.322360082	0.844444444	>= 0.3	Negative	P >= 0.05
Romboutsia	CAT	-0.065262474	0.411111111	< 0.1	Negative	P >= 0.05
Romboutsia	SOD	-0.149347977	0.541666667	0.1 - 0.3	Negative	P >= 0.05
Romboutsia	GSH-Px	-0.068163074	0.370833333	< 0.1	Negative	P >= 0.05
Romboutsia	T-AOC	0.331140423	0.230555556	>= 0.3	Positive	P >= 0.05
Romboutsia	MDA	-0.19059311	0.719444444	0.1 - 0.3	Negative	P >= 0.05
Ligilactobacillus	IgA	0.72956099	0.019444444	>= 0.3	Positive	P < 0.05
Ligilactobacillus	IgG	0.786384237	0.001388889	>= 0.3	Positive	P < 0.05
Ligilactobacillus	IgM	0.538262784	0.080555556	>= 0.3	Positive	P >= 0.05
Ligilactobacillus	IL-2	0.924585309	0.001388889	>= 0.3	Positive	P < 0.05
Ligilactobacillus	IL-4	0.419838014	0.168055556	>= 0.3	Positive	P >= 0.05
Ligilactobacillus	IL-1β	0.207092692	0.206944444	0.1 - 0.3	Positive	P >= 0.05
Ligilactobacillus	TNF-α	0.703246567	0.001388889	>= 0.3	Positive	P < 0.05
Ligilactobacillus	CAT	-0.363653449	0.940277778	>= 0.3	Negative	P >= 0.05
Ligilactobacillus	SOD	-0.348502979	0.836111111	>= 0.3	Negative	P >= 0.05
Ligilactobacillus	GSH-Px	0.02990918	0.311111111	< 0.1	Positive	P >= 0.05
Ligilactobacillus	T-AOC	0.289161587	0.255555556	0.1 - 0.3	Positive	P >= 0.05
Ligilactobacillus	MDA	0.155364618	0.309722222	0.1 - 0.3	Positive	P >= 0.05
Bacteroides	IgA	0.673971643	0.002777778	>= 0.3	Positive	P < 0.05
Bacteroides	IgG	0.479108246	0.0125	>= 0.3	Positive	P < 0.05
Bacteroides	IgM	0.795796086	0.001388889	>= 0.3	Positive	P < 0.05
Bacteroides	IL-2	0.410637928	0.168055556	>= 0.3	Positive	P >= 0.05
Bacteroides	IL-4	0.862943396	0.001388889	>= 0.3	Positive	P < 0.05
Bacteroides	IL-1β	0.853199269	0.001388889	>= 0.3	Positive	P < 0.05
Bacteroides	TNF-α	0.56873776	0.026388889	>= 0.3	Positive	P < 0.05
Bacteroides	CAT	-0.049017564	0.454166667	< 0.1	Negative	P >= 0.05
Bacteroides	SOD	0.242186638	0.230555556	0.1 - 0.3	Positive	P >= 0.05
Bacteroides	GSH-Px	-0.246164965	0.794444444	0.1 - 0.3	Negative	P >= 0.05
Bacteroides	T-AOC	-0.070929125	0.502777778	< 0.1	Negative	P >= 0.05
Bacteroides	MDA	0.2787988	0.152777778	0.1 - 0.3	Positive	P >= 0.05
Bacillus	IgA	0.613845698	0.004166667	>= 0.3	Positive	P < 0.05
Bacillus	IgG	0.492018886	0.015277778	>= 0.3	Positive	P < 0.05
Bacillus	IgM	0.74815431	0.001388889	>= 0.3	Positive	P < 0.05
Bacillus	IL-2	0.646567762	0.004166667	>= 0.3	Positive	P < 0.05
Bacillus	IL-4	0.589044184	0.002777778	>= 0.3	Positive	P < 0.05

Bacillus	IL-1β	0.747822579	0.001388889	>= 0.3	Positive	P < 0.05
Bacillus	TNF-α	0.716605726	0.001388889	>= 0.3	Positive	P < 0.05
Bacillus	CAT	0.013442326	0.497222222	< 0.1	Positive	P >= 0.05
Bacillus	SOD	0.083112899	0.355555556	< 0.1	Positive	P >= 0.05
Bacillus	GSH-Px	-0.262575695	0.841666667	0.1 - 0.3	Negative	P >= 0.05
Bacillus	T-AOC	-0.207552846	0.772222222	0.1 - 0.3	Negative	P >= 0.05
Bacillus	MDA	0.07308057	0.322222222	< 0.1	Positive	P >= 0.05
Lactobacillus	IgA	0.645447997	0.001388889	>= 0.3	Positive	P < 0.05
Lactobacillus	IgG	0.714583983	0.018055556	>= 0.3	Positive	P < 0.05
Lactobacillus	IgM	0.618659772	0.001388889	>= 0.3	Positive	P < 0.05
Lactobacillus	IL-2	0.475257559	0.034722222	>= 0.3	Positive	P < 0.05
Lactobacillus	IL-4	0.781623518	0.001388889	>= 0.3	Positive	P < 0.05
Lactobacillus	IL-1β	0.5372739	0.018055556	>= 0.3	Positive	P < 0.05
Lactobacillus	TNF-α	0.494422608	0.018055556	>= 0.3	Positive	P < 0.05
Lactobacillus	CAT	-0.189358415	0.755555556	0.1 - 0.3	Negative	P >= 0.05
Lactobacillus	SOD	-0.059399742	0.602777778	< 0.1	Negative	P >= 0.05
Lactobacillus	GSH-Px	-0.05099418	0.493055556	< 0.1	Negative	P >= 0.05
Lactobacillus	T-AOC	0.330347116	0.102777778	>= 0.3	Positive	P >= 0.05
Lactobacillus	MDA	0.413634947	0.118055556	>= 0.3	Positive	P >= 0.05
Helicobacter	IgA	-0.163190425	0.734722222	0.1 - 0.3	Negative	P >= 0.05
Helicobacter	IgG	-0.109059584	0.626388889	0.1 - 0.3	Negative	P >= 0.05
Helicobacter	IgM	-0.135331154	0.648611111	0.1 - 0.3	Negative	P >= 0.05
Helicobacter	IL-2	-0.147551321	0.688888889	0.1 - 0.3	Negative	P >= 0.05
Helicobacter	IL-4	-0.122532559	0.684722222	0.1 - 0.3	Negative	P >= 0.05
Helicobacter	IL-1β	-0.260168385	0.905555556	0.1 - 0.3	Negative	P >= 0.05
Helicobacter	TNF-α	-0.034444977	0.563888889	< 0.1	Negative	P >= 0.05
Helicobacter	CAT	0.318404003	0.108333333	>= 0.3	Positive	P >= 0.05
Helicobacter	SOD	0.001045532	0.444444444	< 0.1	Positive	P >= 0.05
Helicobacter	GSH-Px	-0.246195052	0.870833333	0.1 - 0.3	Negative	P >= 0.05
Helicobacter	T-AOC	-0.20955949	0.775	0.1 - 0.3	Negative	P >= 0.05
Helicobacter	MDA	0.529000509	0.041666667	>= 0.3	Positive	P < 0.05
Clostridium_sensu_stricto_1	IgA	-0.263539706	0.7625	0.1 - 0.3	Negative	P >= 0.05
Clostridium_sensu_stricto_1	IgG	-0.266152909	0.847222222	0.1 - 0.3	Negative	P >= 0.05
Clostridium_sensu_stricto_1	IgM	-0.21607025	0.7125	0.1 - 0.3	Negative	P >= 0.05
Clostridium_sensu_stricto_1	IL-2	-0.121891942	0.565277778	0.1 - 0.3	Negative	P >= 0.05
Clostridium_sensu_stricto_1	IL-4	-0.344639436	0.854166667	>= 0.3	Negative	P >= 0.05
Clostridium_sensu_stricto_1	IL-1β	-0.227364204	0.733333333	0.1 - 0.3	Negative	P >= 0.05
Clostridium_sensu_	TNF-α	-0.096813812	0.590277778	< 0.1	Negative	P >= 0.05

stricto_1						
Clostridium_sensu_ stricto_1	CAT	0.689374242	0.005555556	>= 0.3	Positive	P < 0.05
Clostridium_sensu_ stricto_1	SOD	0.281437156	0.208333333	0.1 - 0.3	Positive	P >= 0.05
Clostridium_sensu_ stricto_1	GSH-Px	-0.313683571	0.915277778	>= 0.3	Negative	P >= 0.05
Clostridium_sensu_ stricto_1	T-AOC	-0.51865309	0.995833333	>= 0.3	Negative	P >= 0.05
Clostridium_sensu_ stricto_1	MDA	0.200258931	0.206944444	0.1 - 0.3	Positive	P >= 0.05
Corynebacterium	IgA	0.255660073	0.204166667	0.1 - 0.3	Positive	P >= 0.05
Corynebacterium	IgG	0.202343846	0.1	0.1 - 0.3	Positive	P >= 0.05
Corynebacterium	IgM	0.150215634	0.331944444	0.1 - 0.3	Positive	P >= 0.05
Corynebacterium	IL-2	0.512916556	0.056944444	>= 0.3	Positive	P >= 0.05
Corynebacterium	IL-4	-0.082879693	0.483333333	< 0.1	Negative	P >= 0.05
Corynebacterium	IL-1β	0.043070686	0.430555556	< 0.1	Positive	P >= 0.05
Corynebacterium	TNF-α	0.294818796	0.169444444	0.1 - 0.3	Positive	P >= 0.05
Corynebacterium	CAT	-0.263132267	0.780555556	0.1 - 0.3	Negative	P >= 0.05
Corynebacterium	SOD	-0.445735293	0.966666667	>= 0.3	Negative	P >= 0.05
Corynebacterium	GSH-Px	-0.056289265	0.465277778	< 0.1	Negative	P >= 0.05
Corynebacterium	T-AOC	0.111372233	0.354166667	0.1 - 0.3	Positive	P >= 0.05
Corynebacterium	MDA	-0.128414662	0.648611111	0.1 - 0.3	Negative	P >= 0.05
Rothia	IgA	0.495371861	0.145833333	>= 0.3	Positive	P >= 0.05
Rothia	IgG	0.178639683	0.243055556	0.1 - 0.3	Positive	P >= 0.05
Rothia	IgM	0.296552971	0.256944444	0.1 - 0.3	Positive	P >= 0.05
Rothia	IL-2	0.654198205	0.129166667	>= 0.3	Positive	P >= 0.05
Rothia	IL-4	0.038003471	0.402777778	< 0.1	Positive	P >= 0.05
Rothia	IL-1β	0.054324431	0.359722222	< 0.1	Positive	P >= 0.05
Rothia	TNF-α	0.419188232	0.125	>= 0.3	Positive	P >= 0.05
Rothia	CAT	0.170276846	0.247222222	0.1 - 0.3	Positive	P >= 0.05
Rothia	SOD	-0.04770691	0.491666667	< 0.1	Negative	P >= 0.05
Rothia	GSH-Px	-0.317152347	0.834722222	>= 0.3	Negative	P >= 0.05
Rothia	T-AOC	-0.315190727	0.7625	>= 0.3	Negative	P >= 0.05
Rothia	MDA	0.354789299	0.109722222	>= 0.3	Positive	P >= 0.05
Sphingomonas	IgA	0.437580501	0.0875	>= 0.3	Positive	P >= 0.05
Sphingomonas	IgG	0.494114109	0.034722222	>= 0.3	Positive	P < 0.05
Sphingomonas	IgM	0.20012081	0.306944444	0.1 - 0.3	Positive	P >= 0.05
Sphingomonas	IL-2	0.577712035	0.101388889	>= 0.3	Positive	P >= 0.05
Sphingomonas	IL-4	0.157718425	0.358333333	0.1 - 0.3	Positive	P >= 0.05
Sphingomonas	IL-1β	-0.096315516	0.456944444	< 0.1	Negative	P >= 0.05
Sphingomonas	TNF-α	0.343569096	0.129166667	>= 0.3	Positive	P >= 0.05
Sphingomonas	CAT	-0.497619967	0.990277778	>= 0.3	Negative	P >= 0.05
Sphingomonas	SOD	-0.342838741	0.769444444	>= 0.3	Negative	P >= 0.05

Sphingomonas	GSH-Px	0.420705613	0.318055556	>= 0.3	Positive	P >= 0.05
Sphingomonas	T-AOC	0.353603613	0.172222222	>= 0.3	Positive	P >= 0.05
Sphingomonas	MDA	-0.040851691	0.534722222	< 0.1	Negative	P >= 0.05
